# Magnetic Graphene Oxide Composite for the Microextraction and Determination of Benzophenones in Water Samples

**DOI:** 10.3390/nano10010168

**Published:** 2020-01-18

**Authors:** Alejandro Medina, Francisco Antonio Casado-Carmona, Ángela I. López-Lorente, Soledad Cárdenas

**Affiliations:** Departamento de Química Analítica, Instituto Universitario de Investigación en Química Fina y Nanoquímica IUNAN, Universidad de Córdoba, Campus de Rabanales, Edificio Marie Curie, E-14071 Córdoba, Spain; q42mejaa@uco.es (A.M.); q92cascf@uco.es (F.A.C.-C.)

**Keywords:** dispersive micro-solid phase microextraction, benzophenones, magnetic nanoparticles, graphene oxide, swimming pool water

## Abstract

Magnetite nanoparticles (Fe_3_O_4_) functionalized with graphene oxide (GO) have been synthesized through a silanization process of the magnetic nanoparticles with tetraethyl orthosilicate and (3-aminopropyl)triethoxysilane and further coupling of GO. The synthesized nanomaterials have been characterized by several techniques, such as transmission electron microscopy (TEM), and infrared and Raman spectroscopy, which enabled the evaluation of the different steps of the functionalization process. The hybrid nanomaterial has been employed for the extraction of five benzophenones (benzophenone-1, benzophenone-3, 4-hydroxybenzophenone, benzophenone-6 and benzophenone-8) in aqueous samples by dispersive micro-solid phase extraction, combining the magnetic properties of magnetite nanoparticles with the excellent sorption capacity of graphene oxide via hydrophobic interactions with the analytes. The subsequent separation and quantification of the analytes was performed by liquid chromatography with tandem mass spectrometric detection, achieving limits of detection (LODs) in the range 2.5 to 8.2 μg·L^−1^, with relative standard deviations ranging from 1.3–9.8% and relative recovering in the range 86 to 105%. Positive swimming pool water samples analysed following the developed method revealed the presence of benzophenones in from 14.3 to 39 μg·L^−1^.

## 1. Introduction

Graphene and its derived materials are attracting great attention in the last years in many fields such as electrochemistry, energy storage, membranes for gas transport or water treatment, and microextraction techniques, among others [1,2,3]. Their outstanding properties, such as high specific surface area and the presence of a delocalized π–π system, which provides good affinity towards aromatic compounds, have boosted their application in the microextraction context. However, its broadband application is hindered by the tendency of carbon nanomaterials to aggregation, which has been overcome by the use of the graphene derivative graphene oxide (GO). Graphene can be easily oxidized to GO, which possesses rich oxygen functional groups, such as hydroxyl, epoxy, and carboxyl, and shows good dispersibility in water. GO has been employed in solid phase microextraction (SPME) mainly incorporated to composite materials, e.g., with cross-linked polyoxyethylene as fiber coating material [4], incorporated poly acrylamide-ethylene glycol dimethacrylate monolithic fiber [5], packed in-tube solid phase microextraction supporting GO on aminopropyl silica [6], magnetic nanoparticles (MNPs) combined to reduced GO, and graphitic carbon nitride filled in a polypropylene hollow tube [7]. On the other hand, GO has been also evaluated as a sorbent in the so-called dispersive micro-solid phase extraction (DμSPE) [8], also in hybrid sorbents such as GO and molecularly imprinted polymers [9]. In DμSPE, the sorbent is dispersed into the sample, the close contact favoring the kinetics of the sorption and increasing the efficiency of the overall process [10]. Nevertheless, collection of the dispersed material is usually a bottleneck in detriment of sample speediness, which can be carried out, e.g., by centrifugation; cloud point extraction [11]; or, as proposed herein, by combination of GO with MNPs.

Magnetic nanoparticles possess unique properties which allow them to be guided by simply applying magnetic field. The combination of MNPs with other nanomaterials as well as micrometric systems has increased the potential of this nanomaterial for microextraction. In this sense, MNPs have been combined with micrometric polymers [12,13] or ionic liquids [14], exploiting the extraction capabilities of the polymeric network or ionic liquid at the same time that the magnetic behavior simplifies the overall procedure. Hybrids of cobalt ferrite MNPs coated with oleic acid [15] as well as MNPs with graphitized carbon black [16] have been employed for the determination of UV filters. In addition, different methods have been described for the preparation of hybrids GO-MNPs [17,18,19,20], especially for biomedical applications [21]. Hybrid sorbents of GO with magnetic properties have been also employed for water remediation, e.g., for the removal of atrazine [22] or bisphenol A [23] from the aqueous medium. In the microextraction context, GO has been combined with MnFe_2_O_4_ [24] and ZnFe_2_O_4_ NPs [25] for the extraction of metals. Wang et al. combined Fe_3_O_4_ nanoparticles with GO as nanocomposite adsorbent for the analysis of sulfonamides in milk samples [26] and pesticide determination [27]. These composites are usually prepared by coprecipitation of the MNPs in the presence of GO.

In this work, a hybrid sorbent for DμSPE comprising GO obtained via oxidation of graphene covalently attached to magnetic nanoparticles (Fe_3_O_4_) has been developed and evaluated for the first time for the extraction and determination via liquid chromatography with tandem mass spectrometric detection of endocrine disrupters, i.e., benzophenones. Endocrine disrupting chemicals (EDs) are both natural and synthetic compounds that may affect the normal function of the endocrine system of human being, which are a rising concern due to their negative effects [28]. This term comprises different groups of compounds, such as parabens, bisphenol A and benzophenones [29]. The family of benzophenones is composed by 12 main compounds, so-called benzophenone-1 to benzophenone-12, as well as the 2-, 3-, and 4-hydroxy substituted benzophenones [30]. They are commonly used in sunscreen lotions, as they can act as UV filters, and they are also found in personal care products, these cosmetic ingredients being considered as emerging contaminants as they can easily reach the aquatic environment. Thus, there is a need of developing analytical methods capable of sensitively detect these compounds in aqueous samples in contact with sunscreens and cosmetics. In this context, the so-called microextraction techniques play an important role in sample preparation for the determination of this kind of compounds in environmental samples [31], which are commonly coupled to a subsequent analytical separation technique such as chromatography, due to the complexity of these matrices.

The magnetic nanocomposite was obtained through a multi-step procedure, comprising a first covering with silica, and the subsequent incorporation of amine groups which are covalently coupled to carboxylic groups of GO. The composite sorbent can be easily dispersed in water while the magnetic core of the NPs enables sorbent recovery by application of an external magnetic field via a magnet, overcoming the aggregation problems of carbon nanomaterials and maximizing the interaction with the target analytes. Furthermore, the procedure is simple, as the magnetic composite avoids centrifugation and filtration steps. The excellent adsorption properties of GO are exploited for the efficient extraction of benzophenones via dispersive microextraction technique. Five of the most commonly used benzophenones in personal care products were selected as representative analytes of the benzophenone family. The covalent attachment of GO to the MNPs ensures the stability and reusability of the nanocomposite, without loss of the carbon nanomaterial. The method has been applied to the determination of benzophenones in swimming pool samples.

## 2. Materials and Methods 

### 2.1. Reagents

All the reagents were of analytical grade or better and were purchased from Sigma-Aldrich (Madrid, Spain) unless otherwise is stated. Analytes employed were benzophenone-1 (BP-1), benzophenone-3 (BP-3), 4-hydroxybenzophenone (4-OH-BP), benzophenone-6 (BP-6) benzophenone-8 (BP-8), and oxybenzone-(phenyl-d_5_) (BP-3-d_5_), used as internal standard in mass spectrometry. Stock standard solutions of each of them were prepared in acetonitrile at a 1000 mg·L^−1^ concentration. Working standards at different concentrations were prepared on a daily basis by dilution of the stock solution of each analyte in Milli-Q water (Millipore Corp., Madrid, Spain). In the extraction procedure, sodium chloride was used to adjust the ionic strength while hydrochloric acid and sodium hydroxide were employed for pH adjustment. Methanol was used as eluent. 

Iron (III) chloride hexahydrate (FeCl_3_.6H_2_O), iron (II) chloride tetrahydrate (FeCl_2_·4H_2_O), and ammonia were employed for the synthesis of magnetite nanoparticles. Ethanol, ammonia, tetraethylorthosilicate (TEOS), and methanol were employed for covering the magnetic core of the nanoparticles with a silica layer, whereas 3-aminopropyltrietoxysilane (APTES), toluene, and methanol were used for the subsequent amino-termination of the NPs. 

Graphene (avangrafeno-2, <2 nm and <6 sheets), provided by Avanzare Innovación Tecnológica SL (Logroño, Spain), was oxidized to graphene oxide (GO) by using a mixture of sulfuric acid and nitric acid. N-(3-Dimethylaminopropyl)-N′-ethylcarbodiimide (EDC) and N-hydroxysuccinimide (NHS) were employed for the coupling of GO to the NPs.

### 2.2. Characterization of the Nanomaterials

The characterization of the synthesized materials was carried out by using different microscopic and spectroscopic techniques. Transmission electron microscopy (TEM) images were acquired with a JEOL JEM-1400 microscope (JEOL, Peabody, MA, USA). A drop of the different nanoparticle suspensions was drop-cast on a copper TEM grid with a Carbowax forward.

Infrared spectra were performed with a 37 FT-IR Tensor spectrometer (Bruker Otik, Ettlinger, Germany), equipped with a three internal reflections diamond attenuated total reflection (ATR) cell with a circular surface of 3 mm of diameter (Platinum ATR accessory, Bruker). A room temperature deuterated triglycine sulfate (DTGS) detector was used for spectra acquisition. Spectra were collected in the region 4000 to 400 cm^−1^ with 4 cm^−1^ resolution and accumulating 64 scans. Air spectra were used as background. Data acquisition and treatment were performed via OPUS software (Bruker). 

Raman measurements were carried out with a confocal Raman spectrometer (alpha500, WITec GmbH, Ulm, Germany). A double frequency Nd:YAG laser at a wavelength of 532 nm was employed for excitation. The beam was focused by using a 20× Zeiss objective with a numerical aperture (NA) of 0.4. An electron multiplier charge couple device (EMCCD) was used a detector. Raman spectra were acquired with an integration time of 2 s by accumulating a total of 50 spectra.

Dynamic light scattering (DLS) and Z-potential measurements were performed using a Zetasizer-Nano ZSP (Malvern Instruments, Malvern, UK) operating with a 10 mW 633 nm laser. DLS measurements were performed by using disposable polystyrene cells in backscattering configuration with the detector at a 173° fixed angle. Measurements of Z-potential were performed in a capillary cell using forward scattering setup.

### 2.3. Chromatographic Systems

Two chromatographic systems, including different detectors, were employed in the development of the present research. The optimization of the extraction procedure was carried out on a HP1100 series liquid chromatograph (Agilent, Palo Alto, CA, USA) equipped with a binary high-pressure pump for mobile phase delivery, an autosampler and a single wavelength photometer (HP1100 series). Data analysis was performed using HP ChemStation software. Chromatographic separation was performed with an Ultrabase C18 (250 × 4.6 mm, 5 µm) column (Análisis Vínicos, Tomelloso, Spain). The mobile phase consisted of 0.1% acetic acid aqueous solution (solvent A) and acetonitrile (solvent B) as mobile phase components at flow rate of 1.0 mL·min^−1^ using a gradient elution program. The initial composition was fixed at 55% B, maintained 9 min, after that the percentage being increased up to 65% until 18 min, and finally it returned to the initial conditions in 3 min. The injection volume was 20 μL, and the analytes were determined at 254 nm.

The validation of the methodology was carried out on an Agilent 1260 Infinity HPLC system (Agilent, Palo Alto, CA, USA) equipped with a binary high-pressure pump for mobile phase delivery and an autosampler. Chromatographic separation was performed on an Eclipse XDB-C18 (150 × 4.6 mm, 5 µm) column from Agilent, using 0.1% ammonium formate aqueous solution and acetonitrile as mobile phase components in a 25/75 (*v*/*v*) ratio. A guard column (0.2 µm filter, 2.1 mm), also from Agilent, was used to preserve the integrity of the analytical column. The injection volume was 5 μL and the flow rate was maintained at 0.5 mL·min^−1^.

Quantification was performed on Agilent 6420 Triple Quadrupole MS with electrospray source using Agilent MassHunter Software (Version B.06.00) for qualitative and quantitative analyses. The mass spectrometer settings were fixed to improve the multiple reaction monitoring (MRM) signals. The flow rate and the temperature of the drying gas (N_2_) were 9 L·min^−1^ and 350 °C, respectively. The nebulizer pressure was 30 psi, and the capillary voltage was kept to 3000 V in positive mode. The analytes and the internal standard were detected by MRM transitions, the parameters being specified in Table 1.

### 2.4. Synthesis of GO-Functionalized Magnetic Nanoparticles

The synthesis and functionalization of MNPs was carried out in four steps. First, Fe_3_O_4_ NPs were obtained by a coprecipitation method similar to that previously described [14], and then 24 g of FeCl_3_·6H_2_O and 9.8 g of FeCl_2_·4H_2_O were dissolved in 100 mL of Milli-Q water under nitrogen stream. The solution was stirred for 30 min at 80 °C and then 50 mL of 25 wt% ammonia were added dropwise. The formation of a black precipitate of MNPs is observed, which was recovered with a magnet and washed with water and dried at 80 °C. Once dried, the MNPs were milled to a fine powder.

In the next step, MNPs were coated with silica by mixing 1 g of MNPs with 50 mL of ethanol, 4 mL of Milli-Q water, adjusting the pH to 9.0 with ammonia, and subsequently adding 2 mL of tetraethylorthosilicate (TEOS) [32]. The suspension was stirred for 12 h. Afterwards, the obtained silica-coated magnetic nanoparticles (Fe_3_O_4_@SiO_2_) were recovered with a magnet, washed with Milli-Q water and methanol and dried at 80 °C. Subsequently, Fe_3_O_4_@SiO_2_ were covered with APTES [32]. For this purpose, 400 mg of the Fe_3_O_4_@SiO_2_ were mixed with 1 mL of APTES and 30 mL of toluene in a 3-neck round bottom flask. The mixture was kept under reflux for 48 h under nitrogen stream. Afterwards, the material (Fe_3_O_4_@SiO_2_@APTES) was washed with toluene and methanol and dried at 80 °C.

**Figure 1:**
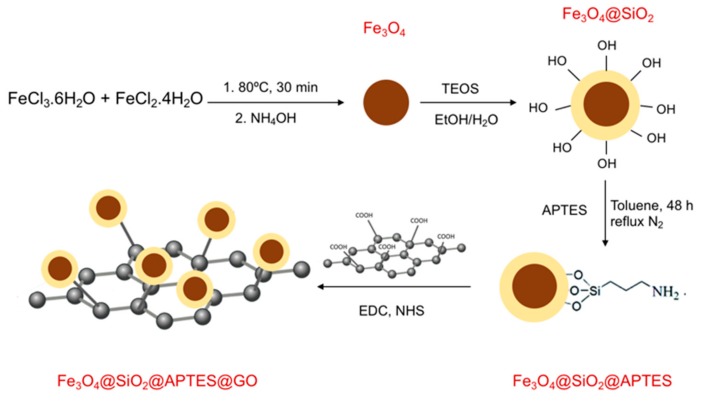
Scheme of the procedure of synthesis and the different steps of the functionalization of MNPs with silica, 3-aminopropyltrietoxysilane and graphene oxide (GO).

The last step of the synthesis comprises functionalization of the material with GO, which had been previously oxidized from graphene. This procedure was carried out by mixing 400 mg of graphene with a 3:1 mixture of sulfuric and nitric acids [33] in a round-bottom flask and refluxing in an ultrasound bath for 45 min. Then, the material was washed and centrifuged, and the obtained GO was left to dry. The immobilization of GO onto Fe_3_O_4_ was carried out using EDC and NHS as coupling agents, in a procedure similar to that described previously [34]. Forty milligrams of the obtained GO was mixed with 200 mg of EDC, 160 mg of NHS, and 60 mL of Milli-Q water, and was kept for 30 min under ultrasound bath. Later, 40 mg of Fe_3_O_4_@SiO_2_@APTES were added and the suspension was submitted to further ultrasound bath energy for 30 min. Afterwards, the solution was stirred in a vortex at 80 °C for 5 h, and it was finally washed with water and dried at 80 °C. Figure 1 depicts a scheme of the different steps of the process of synthesis and functionalization of the material.

### 2.5. Analytical Procedure

First, 100 mL of the sample was adjusted to pH 3 and 4% of NaCl was added. They were then placed in contact with 20 mg of Fe_3_O_4_@SiO_2_@APTES@GO. The mixture was kept for 5 min in ultrasound bath followed by 5 min of vortexing. A vortex stirrer from IKA^®^ (Staufen, Germany) and an ultrasonic bath (50 W, 40 KHz, J.P. Selecta, Barcelona, Spain) were employed in the extraction procedure.

Afterwards, a neodymium magnet (60 mm × 30 mm × 15 mm and a 549.4 M of maximum magnetic force), provided by Supermagnete (Gottmadingen, Germany), was employed for withdrawing the MNPs, thus assisting the removal of the water, and 500 µL of methanol were subsequently added to the MNPs followed by 5 min of sonication. The supernatant was then removed with a syringe while the magnet retains the MNPs, and filtered through a PTFE 0.2 µm (Teknokroma, Barcelona, Spain). After filtration process the supernatant was deposited on a chromatographic vial with insert in order to perform the subsequent identification and quantification of the extracted analytes.

## 3. Results and Discussion

### 3.1. Synthesis and Characterization of the Nanomaterials

Magnetite nanoparticles were chemically synthesized from FeCl_3_·6H_2_O and FeCl_2_·4H_2_O. They were subsequently treated with TEOS through a silanization process. The silica shell on the surface of the MNPs acts as stabilizer, preventing their aggregation and oxidation [32]. Furthermore, the silanol groups at the surface of MNPs can subsequently enable the covalent anchoring of aminopropylsilane groups, which allows the subsequent functionalization with GO via a covalent bond through an amide link between the carboxylic groups of GO and the amine groups exposed in the surface of MNPs. The anchoring of GO at the surface was carried out through a carbodiimide-mediated crosslinking reaction using EDC and NHS as coupling agents, which allow efficient conjugation of –COOH groups to primary amines. The covalent attachment of the GO to the MNPs ensures its stability, preventing GO to be detached from the magnetic core upon recycling of the magnetic sorbent. In fact, after extraction, application of a magnet led to the collection of the whole sorbent material, the solution becoming colorless.

**Figure 2:**
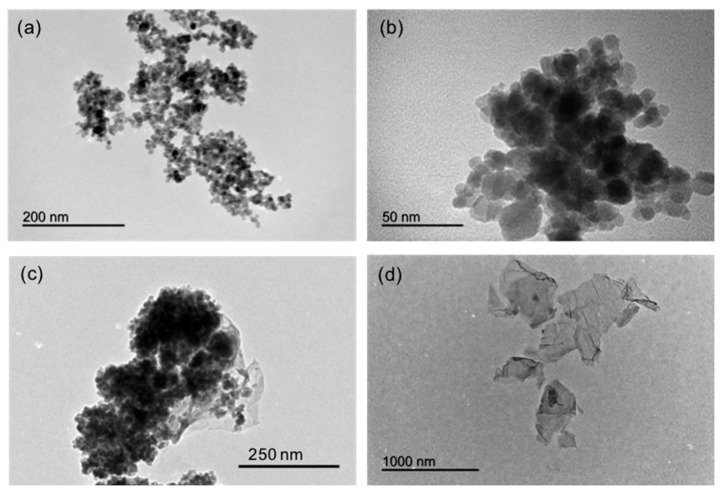
TEM micrographs of the different nanomaterials synthesized: (**a**) magnetic nanoparticles (Fe_3_O_4_), (**b**) magnetic nanoparticles covered with TEOS and APTES (Fe_3_O_4_@SiO_2_-APTES), (**c**) MNPs covered with graphene oxide (Fe_3_O_4_@SiO_2_-APTES@GO), and (**d**) graphene oxide (GO).

The different nanomaterials synthesized were characterized by different techniques, such as transmission electron microscopy (TEM), as shown in Figure 2. Figure 2a depicts a TEM micrograph of the MNPs, which are spherical in shape, with an average diameter of 11 ± 4 nm, and forming small aggregates. Evaluation of the size of the MNPs via dynamic light scattering (DLS) revealed a diameter value (Number PDS analysis) of 19 ± 3 nm with an average Z-potential of 37 ± 1 mV. Figure 2b shows MNPs after covering with silica and APTES, an increase in the size of the functionalized MNPs being observed. A silica layer formed around the aggregates of several MNPs. These were prone to form aggregates upon functionalization because of the magnetic dipolar interaction between magnetite NPs [35]. Such increase in size of the aggregates is depicted also in Figure 2c, once the MNPs have been attached to the GO. A high-density coverage of MNPs on GO surface can be observed. On the other hand, Figure 2d shows a TEM micrograph of the GO sheets obtained via oxidation of graphene sheets in acidic reflux.

**Figure 3:**
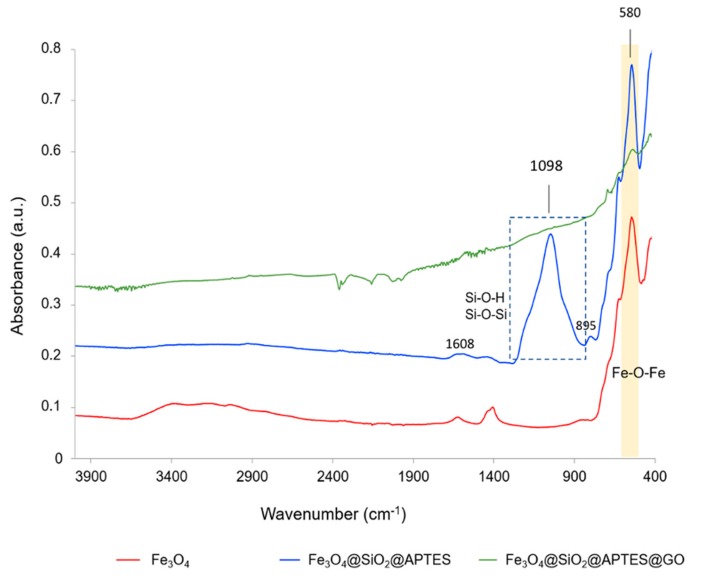
Attenuated total reflectance infrared (ATR-IR) spectra of the different nanomaterials: MNPs; MNPs covered with TEOS and APTES (Fe_3_O_4_@SiO_2_-APTES); and MNPs covered with TEOS, APTES, and graphene oxide (Fe_3_O_4_@SiO_2_-APTES@GO).

The different nanomaterials were also characterized by infrared spectroscopy in attenuated total reflection (ATR) configuration. Figure 3 shows infrared spectra of the different nanomaterials. As can be observed, IR spectrum of MNPs is dominated by a band of 580 cm^−1^, which corresponds to the stretching of Fe–O–Fe bonds. On the other hand, after silanization (Fe_3_O_4_@SiO_2_-APTES) a broad band at approximately 1200–1000 cm^−1^, which can be assigned to the stretching of Si–O–H and Si–O–Si, is observed, which confirms the functionalization of the nanomaterial. A small shoulder at about 895 cm^−1^ could be also assigned to the stretching of Si–O–H. However, the presence of Fe–O–Si bonds cannot be distinguished as it appears at about 584 cm^−1^, which is in the same region than Fe–O vibration of the MNPs [36]. The band about 1608 cm^−1^ can be ascribed to the N–H bending vibration. Regarding the spectrum of MNPs@ SiO_2_-APTES@GO, it resembles similar to that of carbon-based nanomaterials, the signal of Fe–O–Fe from the magnetic core being also slightly seen, while that related to the silanol groups is not visible.

**Figure 4:**
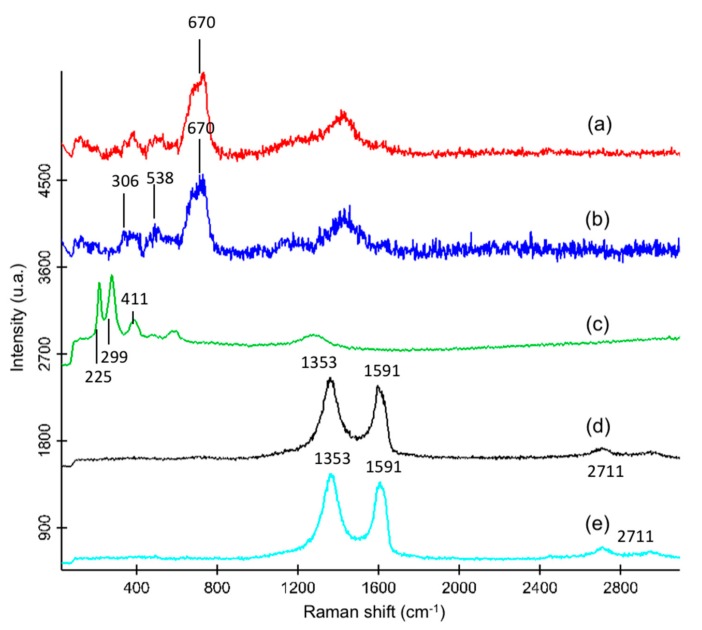
Raman spectra of the different nanomaterials registered with a 532 nm laser at a laser power of 0.08 mW: (**a**) MNPs (Fe_3_O_4_), (**b**) Fe_3_O_4_@SiO_2_-APTES, (**c**) bare MNPs measured with a laser power of 4 mW, (**d**) Fe_3_O_4_@SiO_2_-APTES@GO, and (**e**) GO. Measurement conditions are 2 s of integration time and accumulation of 50 spectra.

Furthermore, nanomaterials were also characterized by Raman spectroscopy, in order to obtain specific spectral features of the attached GO, which were not visible via ATR-IR. Figure 4 shows the Raman spectra of the MNPs at two different laser powers (Figure 4a,c), as well as spectra of Fe_3_O_4_@SiO_2_-APTES (Figure 4b) and Fe_3_O_4_@SiO_2_-APTES@GO (Figure 4d). Finally, Figure 4e depicts the spectrum of GO obtained through oxidation of commercially available graphene powder.

The spectrum of MNPs registered at a laser power <0.1 mW shows a peak at about 670 cm^−1^ (which corresponds to the A_1g_ mode [37]), characteristic of magnetite. It is a relatively broad peak, thus a small contribution of the band at 720 cm^−1^ of maghemite cannot be neglected. Moreover, spectrum in Figure 4a shows also less intense peaks at approximately 306 and 538 cm^−1^, which are in agreement to those described in literature for Fe_3_O_4_ [37]. At these conditions (laser power 0.08 mW), it was not possible to distinguish the silanization process at the surface of the NPs, and no significant differences in spectra of both nanomaterials were found (Figure 4b). Laser power was thus increased up to 4 mW. According to literature, solid samples of magnetite may suffer transformation from magnetite to hematite (*α*-Fe_2_O_3_) as a consequence of the exposure of the sample to laser irradiation, leading to their oxidation. Although the higher value limit of the laser power for the formation of hematite depend on factors such as laser wavelength, objective used, and integration time, some authors describe that the transformation take place at laser power of 1.95 mW [38], whereas other report modification already at laser power as low as 0.4 mW [37]. Figure 4c shows the Raman spectrum of the MNPs acquired at a higher laser power, i.e., 4 mW, observing that the peak at 670 cm^−1^ from Fe_3_O_4_ almost disappear, the spectrum being dominated by hematite bands with peak at about 225 and 299 cm^−1^ [38] as well as 411 cm^−1^. Note that in the spectrum of magnetite nanoparticles registered at low laser power no presence of hematite not formed as a consequence of laser irradiation was detected.

On the other hand, Figure 4d shows the Raman spectrum of Fe_3_O_4_@SiO_2_-APTES@GO, which is dominated by peaks at 1353 and 1591 cm^−1^, which correspond to the so-called D and G bands (tangential mode) of GO, respectively [34]. The spectrum is similar to that observed in the case of GO obtained via acidic treatment from graphene (Figure 4e). Intensity of D band is related to disorder of the graphene sheets and the presence of defects in the crystalline structure which have been intensified upon oxidation process [39] and which are related to the presence of carbon with sp^3^ hybridization. Furthermore, the so-called G’ or 2D band can be also observed at about 2711 cm^−1^, which is related to double resonance phenomena.

### 3.2. Study of the Variables Affecting the Microextraction Process

The variables that affect to the microextraction procedure, i.e., pH, ionic strength, amount of sorbent material per sample volume, agitation, nature of the eluent, and eluent volume, were evaluated in order to achieve the best performance. Their initial values, the interval studied, and the selected values are shown in Table 2. The study was performed in all cases with aqueous standard solutions of 1 mg·L^−1^ of the different benzophenones and their separation and quantification was carried out with a LC-UV system, registering the absorbance at a wavelength of 254 nm versus time. The area of the peaks obtained in the chromatogram corresponding to each analyte was measured for quantitative evaluation of the performance of the method at the different conditions.

**Figure 5:**
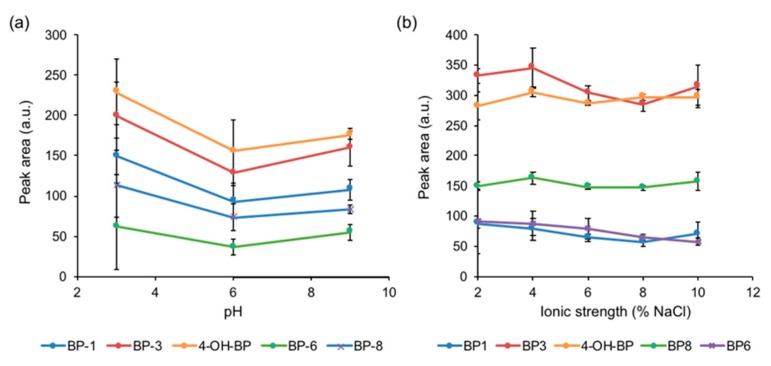
Effect of (**a**) pH and (**b**) ionic strength, expressed as % of NaCl, on the peak area of the analytes calculated from the chromatograms obtained after DμSPE with the developed method at the different conditions and determination via LC-UV.

First of all, pH of the solution was evaluated since it may play an important role during the extraction process, as it determines the charge of the analytes and their interactions with the sorbent. Extractions with standards containing the five benzophenones were carried out in triplicate at different pH values, namely 3, 6, 9, and 12. As can be seen in Figure 5a, pH 3 led to the higher extraction and thus acidic pH values were selected for future measurements. GO possesses carboxylic functional groups at its surface which are protonated at acidic pH values, thus leading to neutral GO, which can promote further hydrophobic interactions with the analytes, such as hydrogen bonds or π–π stacking. In addition, pKa values of benzophenones are in the range of 6.7 to 8.2, thus at acidic pH the hydroxyl groups will be protonated and thus the compounds are neutral thus favoring interaction with the GO nanocomposite sorbent. It should be noted that, as expected, high alkaline pH resulted in damage of the silica core of the functionalized MNPs upon dissolution of the silica, so no values are shown in the figure for that pH value.

Ionic strength may affect the extraction procedure in two different ways. On one hand, it may favor the extraction of the analytes by the so-called salting-out effect. On the other hand, an increase in the viscosity of the sample can diminish the extraction kinetics. Ionic strength was evaluated in the range 0 to 10% (*w*/*v*), using NaCl as model electrolyte, studying the different values in triplicate. As can be observed in Figure 5b, ionic strength did not affect significantly the extraction process, although slightly better extraction efficiency was observed with 4% (*w*/*v*) NaCl, which was then added to the samples.

**Figure 6:**
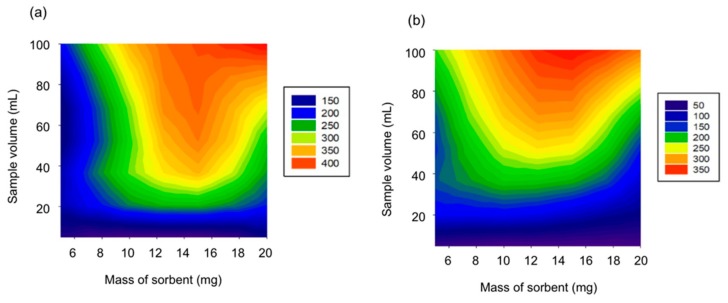
2D curves obtained by plotting the values of peak area (color legend) obtained at different conditions of the two variables evaluated, namely amount of Fe_3_O_4_@SiO_2_-APTES@GO and sample volume for the extraction of (**a**) BP-1 and (**b**) BP-6.

The amount of Fe_3_O_4_@SiO_2_-APTES@GO sorbent and sample volume were evaluated together, by studying the amount of sorbent in the range 5 to 20 mg, while sample volume was investigated in the range of 5 to 100 mL, performing extractions in triplicate. It was observed that for all the analytes the best performance was obtained as expected with the higher sample volume studied. Use of higher volumes was prevented due to practical operational reasons. Nevertheless, three of the analytes (BP-1, 4-OH-BP and BP-8) showed best results with 20 mg of sorbent, while two of them (BP-3 and BP-6) provided better signal with 15 mg of sorbent. Twenty milligrams of sorbent and 100 mL of sample were finally selected, enabling a good dispersion of the Fe_3_O_4_@SiO_2_-APTES@GO and a good interaction with the analytes. Figure 6 depicts the behavior obtained for BP-1 and BP-6 as examples.

To expedite the dispersion of the sorbent in the sample, two different strategies were evaluated, namely, ultrasound bath and vortexing. Different combinations of external energy were assayed, namely, 10, 9, 5, 1, and 0 min of ultrasound completed with vortex agitation until a total time of 10 min. The extraction processes at each condition was carried out in triplicate. It was observed that the combination of 5 min of ultrasound energy followed by 5 min of vortexing agitation resulted in an improved extraction of most of the analytes, thus selecting these conditions.

Moreover, different solvents were investigated in order to evaluate the most suitable for the subsequent elution of the analytes prior chromatographic analysis. Elution was performed with methanol, acetonitrile, and acetone, selecting methanol as it provided the best results. Finally, the volume of eluent was studied in the range 500 to 1000 µL, 500 µL enabling quantitative elution of the retained analytes.

### 3.3. Analytical Figures of Merit

The dispersive micro-solid phase extraction method developed, working under the selected experimental conditions, was characterized in terms of sensitivity, calculating both the limits of detection (LODs) and limits of quantification (LOQs), linearity, and precision. The separation and quantification of the analytes was performed via LC-MS. The values obtained are summarized in Table 3. A calibration graph was built by extracting in triplicate five aqueous solutions of benzophenone mixtures in the range from 5 to 250 µg·L^−1^ and plotting the peak area of the characteristic *m*/*z* fragment ions versus the concentration of each benzophenone. LODs and LOQs were calculated from the linear fit as 3S/N and 10S/N, respectively, obtaining values of LODs ranging from 2.5 µg·L^−1^ for BP-1 to 8.2 µg·L^−1^ for 4-OH-BP, whereas the LOQs were in the range 8.3 µg·L^−1^ and 27.3 µg·L^−1^ for BP-1 and 4-OH-BP, respectively. Linearity was maintained between the LOQ and 250 µg·L^−1^. The correlation coefficients were in the range 0.995 to 0.998 for all analytes. The repetitivity of the method, i.e., intraday precision, expressed as relative standard deviation (RSD), was calculated for five standards containing the target compounds at a concentration of 10 μg·L^−1^, observing a variation between 1.3% (BP-3) and 9.8% (4-OH-BP). Accuracy of the method was evaluated by calculating the relative recoveries. Aqueous samples were spiked at a concentration of 50 µg·L^−1^, achieving values of recovery in the range of 86 to 105%. The reusability of the nanocomposite material was evaluated, being possible to perform the extraction with the same material at least four times without decrease in sensitivity.

### 3.4. Application to Water Samples

The applicability of the developed method was evaluated for the determination of the analytes in swimming pool water samples collected from private use swimming pools in Córdoba. Benzophenones are used in personal care products (such as makeup, hair, and skin care products) and packaging to protect the products from UV lights, and they are commonly used in sunscreens. For example, BP-1 and BP-3 are usually used in nail polishes and lip balms. In 2017 the European Commission reduced the maximum amount of BP-3 allowed in personal care products from 10% *w*/*w* to 6% *w*/*w* in sunscreens, and 0.5% *w*/*w* in other cosmetic products [40]. Two swimming pool samples were analyzed in triplicate by applying the developed method without any pretreatment. Both water samples provided positive results in benzophenones, as summarized in Table 4. As it can be seen, the five benzophenones were detected in the two samples, 4-OH-BP being in both cases below the LOQ, whereas BP-1 was below LOQ in sample 2. Both swimming pool water samples contained higher amount of BP-6 are compared the other analytes. Human exposure to benzophenones has been previously demonstrated, for example BP-1, BP-3, BP-6, and 4-OH-BP were found in samples of human menstrual blood of volunteers in southern Spain, BP-3 being one of the most frequently detected [41]. It has been reported that some of the most used UV filters, including BPs, have been detected even at mg·L^−1^ levels in surface waters [16].

Table 5 shows a comparison with other microextraction methods in which GO has been incorporated, for example, as coating material in fibers for SPME [4,5] or filling materials of hollow tubes [7]. Other authors have reported a magnetic GO composite obtained by performing the coprecipitation method to form the MNPs in the presence of GO [26,27]. In this work, GO has been covalently attached to the surface of MNPs. The covalent attachment of GO to the MNPs ensures stability and reusability of the nanocomposite sorbent material. GO has been incorporated in microextraction techniques for the determination of different organic compounds; nevertheless, as far as we are concern, this is the first time that this combination has been employed for the determination of endocrine disrupters such as benzophenones.

## 4. Conclusions

In this work, a nanocomposite sorbent material comprising magnetite nanoparticles and graphene oxide (Fe_3_O_4_@SiO_2_-APTES@GO) has been developed and applied to the joint determination of endocrine disrupting compounds, i.e., benzophenones. GO was covalently attached to previously amino-terminated MNPs via a coupling reaction forming amide bounds between carboxylic groups of GO and amino groups of MNPs. The different steps of the functionalization procedure were evaluated via vibrational spectroscopy, i.e., infrared and Raman. The synthesized sorbent enabled interaction with the target analytes via hydrophobic interactions with GO, at the same time that the magnetic properties of the Fe_3_O_4_ core of the composite simplifies the overall extraction procedure, enabling collection of the magnetic sorbent via application of an external magnet. The different parameters affecting the extraction procedure were evaluated in order to select the best conditions. With the selected conditions, large sample volumes can be extracted by using small amount of sorbent (20 mg), the whole process being simple and fast. The analytical figures of merit of the method were calculated with standards, obtaining LOQs in the range 8.3 and 27.3 µg·L^−1^, for BP-1 and 4-OH-BP, respectively, allowing, in most cases, their determination in real swimming pool water samples. The five different benzophenones were identified in the two samples, BP-6 being found at higher concentrations.

## Figures and Tables

**Table 1:** Transitions and optimized potentials for LC-MS/MS analysis.

Compound	Precursor Ion (*m*/*z*)	Fragmentor Voltage (V)	Product Ion (*m*/*z*)	Collision Energy (V)	Identification Transition	Quantitation Transition
BP-1	215	115	137	20	215 → 105	215 → 137
			105	20		
BP-3	229.1	125	151	20	229.1 → 105	229.1 → 151
			105	20		
BP-6	275	120	151	20	275 → 95.1	275 → 151
			95.1	45		
BP-8	244.9	110	151	20	244.9 → 151	244.9 → 121
			121	20		
4-OH-BP	199	105	121	20	199 → 77.2	199 → 121
			77.2	45		
BP-3-d_5_	234	135	150.9	20	234 → 110	234 → 150.9
			110	25		

**Table 2:** List of the variables studied affecting the microextraction process, including the initial conditions, the interval evaluated and selected values.

Variable	Initial Value	Interval Studied	Selected Value
pH	Not adjusted	3–12	3
Ionic strength (%NaCl, w/v)	0	0–10	4
Nanocomposite amount (mg)	10	5–20	20
Sample volume (mL)	20	5–100	100
Type of agitation	Mechanical	Mechanical, ultrasounds	Ultrasound + mechanical
Eluent	Methanol	Methanol, acetone and acetonitrile	Methanol
Eluent volume (µL)	500	500–1000	500

**Table 3:** Analytical figures of merit of the developed method comprising DμSPE and LC-MS.

Analyte	LOD (µg·L^−1^)	LOQ (µg·L^−1^)	R^2^	RSD Intra-Day, n = 5 (% RSD)	Accuracy (% RR ^a^)
BP-1	2.5	8.3	0.996	2.4	90 ± 2
BP-3	2.9	9.7	0.998	1.3	105 ± 2
BP-6	7.0	23.3	0.995	6.4	102 ± 7
BP-8	4.0	13.3	0.997	4.3	86 ± 4
4-OH-BP	8.2	27.3	0.995	9.8	93 ± 9

^a^ RR, relative recovery at 50 µg·L^−1^.

**Table 4:** Determination of benzophenone in real swimming pool samples. The results as expressed as mean concentration (in µg·L^−1^) ± standard deviation.

Sample	BP-1	BP-3	BP-6	BP-8	4-OH-BP
Swimming pool 1	28.7 ± 0.7	33.2 ± 0.4	39 ± 2	23 ± 1	Detected
Swimming pool 2	Detected	14.3 ± 0.2	25 ± 2	16.2 ± 0.7	Detected

**Table 5:** Comparison of the proposed method with other reported in the literature incorporating GO in microextraction procedures for the determination of organic compounds.

Incorporation of GO	Extraction Technique	Analyte	Sample	Analytical Technique	LOD	RR (%)	Ref.
GO incorporated in POE ^1^ as fiber coating material	SPME	Phenols	River water	(HS)-SPME-CG-MS	0.12–1.36 ng·L^−1^	81–113	[4]
GO in polymer monolithic fiber	SPME	Organophosphate esters	Soil	HS-SPME-GC/FPD	0.03–0.24 ng·g^−1^	80.1–105.6	[5]
GO supported aminopropyl silica	Packed in-tube SPME	Triazines	Ground and mineral water	HPLC–MS/MS	1.1–2.9 ng·L^−1^	90.8–111	[6]
Fe_3_O_4_@rGO-g-C_3_N_4_ filled in propylene hollow tube	MSPE ^2^	Chlorophenols	Toner	HPLC-UV	0.2–0. 3 μg·Kg^−1^	80.5–104	[7]
GO-MIPs ^3^	DμSPE	Bis(2-ethylhexyl) phthalate	Environmental water	HPLC-UV	0.92 μg·L^−1^	82–92	[9]
Fe_3_O_4_@GO as adsorbent (coprecipitation)	MSPE	Sulfonamides	Milk	HPLC-MS/MS	0.02–0.13 μg·L^−1^	73.4–97.4	[26]
Magnetic graphene oxide (coprecipitation)	SPE + DLLME	Pesticides	Fruit juice and pulp	GC-FID	1–6 μg·Kg^−1^	69–81	[27]
Fe_3_O_4_@SiO_2_@APTES@GO	DμSPE	Benzophenones	Swimming pool water	HPLC-MS/MS	2.5–8.2 μg·L^−1^	86–105	This work

^1^ POE: polyoxyethylene; ^2^ MSPE: magnetic micro solid phase extraction; ^3^ MIPs: molecularly imprinted polymers.
